# BIOKINETICS IN ACHILLES TENDINOPATHY: ESSENTIAL FINDINGS AND CLINICAL APPLICATIONS

**DOI:** 10.1590/1413-785220253306e291432

**Published:** 2025-11-10

**Authors:** Leonardo Metsavaht, Felipe F. Gonzalez, Talissa Oliveira Generoso, Lucas Valério Pallone, Eliane Celina Guadagnin, Alexandre Leme Godoy-Santos, Gustavo Leporace

**Affiliations:** 1 Instituto Brasil de Tecnologias da Saude (IBTS), Rio de Janeiro, RJ, Brazil.; 2 Universidade Federal de Sao Paulo (UNIFESP), Sao Paulo, SP, Brazil.; 3 Rush University Medical Center, Chicago, IL, USA.; 4 Universidade de Sao Paulo, Faculdade de Medicina, Hospital das Clinicas (HCFMUSP), Instituto de Ortopedia e Traumatologia, Sao Paulo, SP, Brazil.

**Keywords:** Tendinopathy, Biomechanics, Kinematics, Motion Analysis, Rehabilitation, Tendinopatia, Biomecânica, Cinemática, Análise de Movimento, Reabilitação

## Abstract

The Achilles tendon, though the strongest in the human body, is the most commonly ruptured and frequently affected by tendinopathy, particularly in athletes. Achilles tendinopathy (AT) impacts approximately 8% of sports participants, with a lifetime incidence of over 50% in runners. Characterized by pain and tenderness, AT significantly compromises quality of life and functional performance. This narrative review explores biomechanical factors contributing to AT, focusing on both kinematic and kinetic parameters and their clinical relevance, providing a review of AT biomechanics literature, nonoperative interventions, and exercises targeting specific biomechanical risks. Studies have linked abnormal motion to AT. Key biomechanical factors include decreased plantar flexion strength, reduced gluteus medius and maximus activity, decreased peak ankle dorsiflexion, altered peak knee flexion, and decreased forward progression of the center of force, which may increase mechanical load and microtrauma, ultimately resulting in tendon damage. The effectiveness of various interventions was examined, emphasizing the integration of specific exercises aimed at addressing distinct biomechanical deficits. Effective management of AT requires addressing strength deficits and biomechanical abnormalities. Traditional rehabilitation protocols focus on strengthening but often neglect critical biomechanical issues. This review highlights the importance of incorporating specific exercises targeting kinematic and kinetic deficiencies. ***Level of Evidence V; Expert Opinion***.

## RESUMO

O tendão de Aquiles, embora seja o mais forte do corpo humano, é também o mais comumente rompido e frequentemente acometido por tendinopatia — especialmente em atletas. A tendinopatia do Aquiles (TA) afeta aproximadamente 8% dos participantes de esportes, com uma incidência ao longo da vida superior a 50% entre os corredores. Caracterizada por dor e sensibilidade, a TA compromete significativamente a qualidade de vida e o desempenho funcional. Esta revisão narrativa explora os fatores biomecânicos que contribuem para a TA, com foco em parâmetros cinemáticos e cinéticos e sua relevância clínica, além de apresentar uma revisão da literatura sobre a biomecânica da TA, intervenções não-operatórias e exercícios direcionados aos riscos biomecânicos. Estudos têm relacionado padrões anormais de movimento à TA. Os principais fatores biomecânicos incluem: diminuição da força de flexão plantar, redução da atividade dos músculos glúteo médio e máximo, redução do pico de dorsiflexão do tornozelo, alteração do pico de flexão do joelho e diminuição da progressão anterior do centro de força — fatores que podem aumentar a carga mecânica e a ocorrência de microtraumas, resultando, por fim, em lesão do tendão. A eficácia de várias intervenções foi examinada. O manejo eficaz da TA exige o tratamento de déficits de força e anormalidades biomecânicas. Protocolos de reabilitação tradicionais focam no fortalecimento, mas frequentemente negligenciam questões biomecânicas críticas. Esta revisão destaca a importância de incorporar exercícios específicos que abordem disfunções cinemáticas e cinéticas. ***Nível de Evidência V; Opinião de Especialista***.

## INTRODUCTION

Despite being the strongest and thickest tendon in the human body.^1^ capable of enduring substantial stresses and forces, the Achilles tendon is the most commonly ruptured tendon and one of the most frequently affected by tendinopathy.^2^ Achilles tendinopathy (AT) affects approximately 8% of individuals involved in sports and physical activities and is one of the most common overuse injuries among runners^3^ with a cumulative lifetime incidence of more than 50% in this population.^4^ Characterized by pain, tenderness to palpation, and thickness, AT can persist for years, causing loss of quality of life and functional capacity.^5,6^

AT has a multifactorial etiology involving diverse intrinsic and extrinsic risk factors. Extrinsic factors associated with AT include the use of quinolones and specific factors related to sports practice such as footwear, training surfaces, changes in training habits, pace, technique and stretching habits^5,7,8^ while intrinsic factors may include vascularity, age, sex, body weight, height, pes cavus deformity, and lateral ankle instability.^5,7^ Several biomechanical aspects have been linked to the genesis and persistence of AT^5,7,9,10^ and understanding each of these biomechanical parameters is essential for effectively treating and rehabilitating patients with AT.

AT mechanism of injury is primarily attributed to overuse.^11,12^ Repetitive microtrauma from mechanical loading leads to tissue damage, which, over multiple loading cycles, impairs tissue function. It has been demonstrated that when the rate of tissue damage surpasses the rate of tissue repair, there is a progressive change in tendon structure. Initially, collagen fibers deform, the interfiber space widens, and ultimately, severe matrix disruption with fiber thinning occurs in fatigue-loaded tendons.^13^ The influence of biomechanical risk factors in AT pathogenesis is due to their contribution to increased mechanical load and microtrauma during motion. When a specific movement pattern increases the mechanical load on the tendon, the likelihood of tissue damage and impaired function increases.^14^

In this context, we present a comprehensive review of the biomechanical factors related to AT and provide insights into their clinical implications. We delve into nonoperative interventions and explore the authors’ preferred exercises tailored to specific biomechanical risk factors.

### Kinematic Parameters

Abnormal kinematic parameters influencing AT, mainly related to rearfoot motion, have been explored by several studies, though with some conflicting results. Many studies have identified specific patterns in patients with AT, such as a greater rearfoot inversion at heel strike, followed by increased peak eversion, a shorter time to maximum eversion, and greater peak eversion velocity through midstance.^9,15-17^ These findings suggest that increased rearfoot eversion could intensify strain on the Achilles tendon, indicating the role of rearfoot motion in AT etiology.

It has been hypothesized that higher rearfoot inversion at initial contact leads to subsequent increased pronation.^16^ inducing inevitable internal tibial rotation. This rotation pulls the Achilles tendon medially, generating a whipping or bowstring effect. The enhanced whipping action could contribute to microtears, especially in the tendon's medial aspect.^16^ Furthermore, pronounced pronation indicates insufficient foot rigidity during stance, necessitating additional effort from extrinsic and intrinsic musculature to stabilize the foot during gait.^7^ These factors may contribute to the overload of the Achilles tendon.^18^

Literature presents controversial results on this parameter. Recently, Mousavi et al. published a meta-analysis reporting that, among kinematic factors, only moderate evidence suggested significant differences between runners with AT and controls for rearfoot eversion at heel strike in shod conditions.^18^ Supporting these findings, Ryan et al., when analyzing barefoot running in AT subjects, detected greater rearfoot eversion during midstance compared to controls, with a trend towards a greater overall range of rearfoot motion.^19^ In contrast, Creaby et al. did not find significant differences in rearfoot eversion peak and range of motion (ROM) in AT subjects during running^20^ and Donoghue et al. also did not find significant differences for a greater rearfoot eversion in AT patients.^21^ However, Donogue et al. found statistical significance when this parameter was exacerbated in shod trials.^21^ Interestingly, in Becker et al.'s study, AT patients did not present greater excursion or velocity of rearfoot eversion compared to controls, but a longer duration of rearfoot eversion was observed.^19,22^ The discrepancies in findings suggest that rearfoot eversion in AT patients may be influenced by factors such as footwear and running conditions.

In terms of other lower limb joint kinematic parameters, there is evidence showing that proximal and distal joints may play a role in AT pathology.^20^ Bramah et al. found that runners with AT demonstrated greater contralateral pelvic drop and forward trunk lean at midstance.^23^ Greater contralateral pelvic drop has been associated with increased rearfoot eversion in a previous study.^24^ It was proposed that this proximal motion in the hip could be inducing rearfoot eversion as a compensatory strategy. However, another study found no evidence of the influence of hip kinematics in runners with AT compared with healthy controls showcasing that this relationship may hold true for certain populations only^20^

A recent meta-analysis by Mousavi et al. found no significant differences between runners with AT and controls for peak knee flexion in both shod and barefoot conditions, as well as for knee flexion ROM in barefoot conditions, although there was conflicting evidence for shod conditions.^18^ Bramah et al. observed a more extended knee and dorsiflexed ankle at initial contact than controls.^23^ Azevedo et al. reported that range of knee flexion between heel strike and midstance during running was significantly lower in patients with AT^25^ similar to findings by Hein et al. and Joachim et al., who observed reduced maximal knee flexion during running in subjects who developed AT.^17,26^ Conversely, Donoghue et al. affirmed that AT patients had greater knee flexion during stance while running.^21^ This variability may be influenced by the fact that insufficient knee flexion is often associated with a protective mechanism to avoid pain, while increased knee flexion can lead to excessive ankle dorsiflexion, increasing tension on the Achilles tendon. These conflicting results underscore the complexity of knee mechanics in AT patients and suggest that further research is needed to better understand the variability and underlying factors influencing knee flexion in this population.

It is important to note that only Hein et al. and Van Ginckel et al. adopted a prospective design^17,27^ making them the only studies whose results allow for inferences of causality. In terms of AT prevention and rehabilitation, it is advised to focus on decreased peak dorsiflexion, decreased peak knee flexion and decreased forward progression of the center of force as these variables are biomechanical risk factors for AT onset and possibly progression.

Moreover, the controversial findings on kinematic characteristics in cross-sectional studies may indicate large differences between AT individuals. Subgroup analysis may be beneficial in this population to better understand the role of biomechanics in the AT etiopathogenesis. Different factors may combine to increase the load on the Achilles tendon, and since not all of them will be present in every individual, biokinetic evaluation is essential to identify which factors each patient possesses, allowing for individualized treatment.

### Kinetic Parameters

Some studies have reported important findings regarding the kinetic parameters of lower limb joints in subjects with AT, with direct implications for clinical treatment and rehabilitation. Kim et al. showed that the internal plantar flexion moment (muscle forces to produce plantar flexion) of AT subjects was reduced from the midstance phase to terminal stance compared to controls, and the internal ankle dorsiflexion moment (muscle forces to produce dorsiflexion) was reduced during the terminal swing phase.^28^ The reduced muscle force production in the ankle during crucial phases of gait means that AT patients may have difficulty pushing off effectively when running, probably leading to compensations in other joints.^28^

Joachim et al. noticed altered knee moments in individuals with AT.^26^ They found reduced knee internal extensor moments during stance in patients with AT. Creaby et al. also reported significant findings regarding hip kinetics.^20^ The authors reported that runners with AT showed an increased peak hip external rotation moment (external joint moment) and impulse compared to controls, as well as a higher hip adduction moment impulse.^20^ These alterations underscore the importance of assessing the entire kinetic chain in patients with AT, as compensations at the knee or hip could contribute to ongoing tendon stress or other injuries.

Studies have also reported on ankle and tibia kinetics. Azevedo et al. and Creaby et al. did not find differences in ankle kinetic parameters,^20,25^ while Williams et al. reported that patients with AT history had a lower external rotation moment of the tibia.^29^ This finding may be due to decreased function of the muscles primarily responsible for transverse-plane motion such as posterior tibialis, resulting in greater strain on the Achilles tendon in the transverse-plane.

Regarding ground reaction forces (GRF), Azevedo et al., Joachim et al., McCrory et al. and Andere et al. did not find differences in the vertical GRF during running between runners with AT and healthy controls.^9,25,26,30^ Lalumiere et al. compared GRF between the symptomatic and asymptomatic limbs of AT patients and found limited differences in total GRF symmetries between the lower limbs.^31^ GRF forces may not be an important parameter to differentiate AT subjects and healthy subjects, and more investigation is crucial to assess the role of this variable as a risk factor before the onset of disease symptoms.

Evidence is also limited regarding plantar pressure distribution during running in patients with AT as few studies report data on this parameter and the results are controversial. Baur et al. demonstrated that patients with AT showed a more medial forward roll in the rear and midfoot than controls, suggesting more pronation in these patients during midstance,^32^ while, Van Ginckel et al. reported an association of AT with a more lateral foot roll-over following heel strike.^27^ Clinically, this suggests the need for individualized assessments of foot mechanics, as variations in foot strike patterns could influence Achilles tendon load and guide personalized interventions, such as orthotic support or gait retraining.

In summary, the kinetic parameters observed in individuals with AT indicate complex and varied alterations in lower limb joint moments and forces. Reduced internal moments at the ankle, knee, and hip during different phases of gait suggest that AT patients may compensate for the reduced function of the Achilles tendon by redistributing forces across other joints. Strengthening the muscles involved in plantar flexion, as part of a force-sharing strategy, is crucial in treatment. Clinically, these findings emphasize the necessity of targeted rehabilitation programs that not only address Achilles tendon deficits but also optimize the kinetic chain to prevent overloading compensatory structures. By identifying these compensatory patterns through individualized kinetic and kinematic assessments, clinicians can tailor interventions to enhance force-sharing mechanisms, ultimately reducing strain on the Achilles tendon and preventing further injury.

### Foot Strike Patterns During Running

Foot strike patterns may impact the magnitude of load on the Achilles tendon during running, potentially influencing the development of injuries. Almonroeder, Willson and Kernozek demonstrated, with weak evidence, that non-rearfoot striker runners exhibited approximately a 15% increase in Achilles tendon loading rate when compared to rearfoot strikers, as well as an 11% higher Achilles tendon impulse in each step.^33^ Similarly, Altman and Davis, in a prospective comparison of shod and barefoot runners, showed that barefoot runners experienced more Achilles tendon and calf injuries, likely due to the midfoot/forefoot strike pattern observed in nearly 80% of barefoot runners.^34^ This strike pattern places greater eccentric demands on the Achilles tendon as the foot dorsiflexes and everts in early stance,^34^ highlighting the need to appropriately strengthen the gastrocnemius-soleus complex in this population.

### Muscle strength

A recent meta-analysis by McAuliffe et al. investigated plantar flexion strength in patients with AT, reporting that subjects with AT demonstrated weaker plantar flexors compared to the uninjured side or healthy controls, showing deficits specifically in maximal, reactive, and explosive strength.^35^ Conversely, another meta-analysis by Hasani et al. found only moderate evidence of impairments in maximal plantar flexor torque and limited evidence for impairment in concentric endurance on the affected side of AT subjects.^36^ There was conflicting evidence for other plantar flexor function, such as explosive strength, power, and other endurance measures, between the affected and unaffected sides and for all measures when compared with healthy controls.^36^

Several studies have identified reduced plantar flexor strength as a prevalent characteristic in individuals with AT and a risk factor for AT, as demonstrated in longitudinal studies. Mahieu et al. demonstrated that male military recruits with weaker plantar flexors developed more injuries during their military training,^37^ and McCrory et al. also observed insufficiency in the gastrocnemius-soleus complex of AT patients, who were recreational or competitive runners, compared to healthy controls.^9^ Masood et al. showed that maximal plantar flexor force was approximately 14% higher in the contralateral limb compared to the AT limb of recreational athletes.^38^ Andere et al. found that plantar flexors exhibited lower total work in runners with AT than in healthy runners.^30^ O’Neill et al. reported that runners with AT had large deficits in plantar flexor torque and endurance with the knees both extended and flexed compared to controls,^39^ and Crowley et al. demonstrated that active individuals with AT had lower maximal plantar flexor strength and power tested with the knee flexed,^40^ suggesting a greater loss of the soleus force-generating capacity rather than the gastrocnemius in AT patients.

Conversely, Sara et al. found no deficits in plantar flexion strength in AT patients, whether evaluated isometrically, concentrically, or eccentrically.^41^ Child et al. and Chimenti et al. did not find differences in plantar flexor strength between AT patients and controls with both the knee flexed^42^ and extended,^43^ respectively. However, Child et al. reported that runners with AT had higher Achilles tendon aponeurosis strain than healthy subjects.^42^

Additionally, Hein et al. observed reduced knee flexor strength in runners that developed AT,^17^ relating it to the genesis of knee stability deficiencies, which may impact lower limb biomechanics and transfer more stress to the Achilles tendon.

In brief, there is a reasonable quantity of studies demonstrating that reduced plantar flexor strength plays a role in the development of AT and possibly maintenance as evidenced by longitudinal and cross-sectional studies. Corroborating this hypothesis, the most commonly used rehabilitation protocols focus on plantar flexor strengthening and show satisfactory results in over 80% of patients.^44^

### Muscle Activity and Neuromuscular Control

Altered muscle activity and neuromuscular control may also play an important role in AT. Smith et al. observed delayed onset and shorter duration of gluteus medius and maximus activation during running in runners with AT compared to healthy individuals, suggesting altered neuromuscular control at the hip level.^45^ This could lead to increased hip adduction and internal rotation, generating greater tibial internal rotation and consequently rearfoot eversion, suggesting a link between hip and ankle biomechanics. Furthermore, a shorter duration of gluteus maximus activation could result in reduced hip extensor power and impaired forward propulsion of the center of mass.^45^ To compensate for the decrease in forward propulsion, the AT could be overloaded in the terminal stance, which is the phase where coordinated contraction between the hip extensors and the plantar flexors is crucial. Additionally, Habets et al. reported that AT individuals demonstrated around 30% less hip isometric abduction strength, less hip isometric external rotation strength, and less hip isometric extension strength in the affected limb, with similar deficits observed in the contralateral healthy limb.^46^ These findings suggest that dysfunction of proximal hip musculature could be associated with increased loading of the distal structures, such as the Achilles tendon, during sport activities.

Azevedo et al. showed a significant decrease in pre-heel strike activity of the tibialis anterior during running in AT runners, as well as post-heel strike activity of the rectus femoris and gluteus medius,^25^ suggesting that runners with AT had a lower capacity than runners free from injuries for shock absorption due to the reduced muscle activation. Conversely, Baur et al. did not identify differences in tibialis anterior activity during running between patients and controls, but observed lower peroneal muscle activation in AT subjects during the weight acceptance phase, as well as reduced gastrocnemius muscle activity during weight acceptance and push-off phases compared to controls.^47^ It is unclear whether lower gastrocnemius activity in AT patients is a risk factor only or also the result of the injury, but the association of AT with mechanical deficits of the lower limb might impair joint stability during the stance phase.^47^

At the distal level, Wyndow et al. demonstrated that runners with AT presented altered triceps surae neuromotor control during running compared to healthy subjects, showing earlier activation of the soleus.^48^ This imbalance could be related to altered intra-tendinous loads in AT. Furthermore, Crouzier et al. reported a lower contribution of the gastrocnemius lateralis to the overall triceps surae activation in individuals with AT compared to controls during maximal and submaximal isometric plantarflexion tasks, with gastrocnemius lateralis contributing 28% less to the total triceps surae force in AT subjects.^49^ These findings suggest differences in force-sharing strategies within the triceps surae in AT patients compared to controls.

### Ankle Flexibility

Limited ankle dorsiflexion passive ROM is another factor associated with AT. Several studies have shown a significant correlation between AT and tightness in the gastrocnemius or soleus muscles. ^16,50-52^ However, some authors suggest that this limitation may not be clinically relevant^51^ or may be nonexistent during physical examination.^17^

Hein et al. and Joachim et al. reported lower peak ankle dorsiflexion during running in AT patients compared to controls.^17,26^ Ryan et al. found a trend toward lower peak ankle dorsiflexion velocity in AT patients.^19^ Conversely, Ferreira et al. did not find an association between ankle dorsiflexion and the occurrence of AT,^53^ and Creaby et al. did not find differences in dorsiflexion parameters during running between patients and controls.^20^ Donoghue et al. reported greater dorsiflexion during running in AT patients compared to controls,^15^ suggesting a complex association between passive and active dorsiflexion ROM and AT, which may underlie the presence of distinct motion-based subgroups.

Ankle dorsiflexion ROM significantly influences lower limb mechanics, especially during tasks that require a high ROM, such as landing. Limited dorsiflexion ROM can lead to compensatory changes in lower limb kinematics, including increased ankle and foot pronation, knee valgus, and increased landing forces.^54^ Furthermore, restricted ROM has been associated with altered gait patterns, such as increased ankle abductor moments and knee flexor and internal rotator moments during the stance phase,^55^ potentially increasing the risk of injuries in athletes.

### Biokinetic Variability

AT is a condition with important biomechanical variability. This is reflected in conflicting evidence concerning kinematic and kinetic parameters, muscle strength, flexibility and neuromuscular activation. Table 1 summarizes the main biomechanical parameters reported across studies, highlighting some of the controversies between them. The presence of substantial heterogeneity identified in this population demonstrates the importance of a personalized approach to treatment.

**Table 1:** Main Biokinetic Characteristics of Patients with Achilles Tendinopathy.

Rearfoot Kinematics	– Greater rearfoot inversion at heel strike – Increased peak eversion – Shorter time to maximum eversion – Greater peak eversion velocity during midstance – Controversy in studies, but shod conditions often show increased rearfoot eversion in AT subjects. Some studies find no differences in eversion in barefoot conditions. – Longer duration of rearfoot eversion in some AT patients.
Knee Kinematics	– Conflicting evidence on knee flexion ROM during running – Reduced maximal knee flexion observed in some studies, while others report greater knee flexion during stance. – AT patients often demonstrate altered knee mechanics, potentially linked to pain avoidance or compensatory strategies.
Ankle Kinematics	– Prospective studies report lower peak ankle dorsiflexion in AT patients compared to controls. – Conflicting evidence on whether limited ankle dorsiflexion ROM is associated with AT. – Complex association between passive and active dorsiflexion ROM and AT, suggesting the presence of distinct subgroups.
Hip Kinematics	– Greater contralateral pelvic drop and forward trunk lean in AT patients. – Some studies found no differences in hip kinematics between AT patients and controls.
Kinetic Parameters	– Reduced plantar flexion and dorsiflexion moments at the ankle in AT patients, leading to difficulty in push-off during gait. – Increased knee internal extensor moments during stance. – Altered force distribution across joints as compensatory mechanisms. – Conflicting evidence on ground reaction forces (GRF).
Muscle Strength	– Weaker plantar flexors in AT patients compared to healthy controls. – Reduced maximal, reactive, and explosive strength in the plantar flexors. – Some studies find no significant strength deficits in AT patients. – Reduced knee flexor strength in AT patients, possibly influencing lower limb biomechanics and increasing Achilles tendon stress.
Muscle Activity	– Delayed and reduced gluteus medius and maximus activation during running in AT patients. – Reduced activity of the gastrocnemius and peroneal muscles during running. – Altered triceps surae neuromotor control, with earlier activation of the soleus. – Differences in force-sharing strategies within the triceps surae in AT patients compared to controls.
Foot Strike Patterns	– Midfoot/forefoot strike pattern during running in non-rearfoot strikers is associated with higher Achilles tendon loading. – Barefoot runners (who often use a midfoot strike) experience more Achilles tendon injuries.
Ankle Flexibility	– Limited ankle dorsiflexion passive ROM is associated with AT in some studies. – Other studies report no significant differences in ankle dorsiflexion in AT patients.

A biokinetic analysis—an assessment that comprises three-dimensional motion analysis, strength and flexibility test - when employed as a diagnostic clinical tool, is critical in the context of AT. Identifying biomechanical risk factors at an individual level can provide substantial information to personalized treatment regimens. Targeting individual-specific dysfunctions, as opposed to generic interventions, may provide faster, cheaper, and more effective treatment.

There is evidence that gait retraining strategies, which include strength, flexibility, neuromuscular, and biofeedback interventions, can be effective in addressing gait alterations.^56,5758^ However, no consensus exists regarding the optimal treatment for biomechanical risk factors associated with AT.^59^

### Treatment

The management of AT involves minimizing pain, allowing the tendon to repair from repetitive damage, and restoring the tendon's capacity to support load.^11,60^ Interventions for AT are diverse and can involve exercises, injections, shockwave therapy, orthosis, acupuncture, medications, and surgery. The initial treatment is usually nonoperative, with surgical options reserved as a last resourt.^61^ However, there is no consensus on the best intervention for this condition.

A recent systematic review with randomized clinical trials has shown that intervention modalities like exercise therapy, injection therapy, shockwave therapy, acupuncture (and combinations of these modalities) are better than "wait-and-see" approach at reducing pain levels at 3 months.^62^ These results indicate that "wait-and-see" therapy is not clinically recommended and should not be considered ethical for future studies. Exercise combined with shockwave therapy and acupuncture alone was superior to most other treatments at 3 months, including exercise therapy alone. At 12 months, there was no difference between treatment modalities, which included exercise therapy, injection therapy, exercise combined with injection therapy, and exercise combined with night-splint therapy.

Most patients respond well to initial management within one year; however, about 20% do not improve.^44^ For these patients, AT issues may persist for over 10 years, leading to a loss of quality of life and impairing daily activities, heavy work, and sports practice.^63^ A potential reason for this non-optimal outcome is that biomechanical variables associated with AT, such as the kinematic and kinetic parameters and neuromuscular control reported in this review are not addressed by the current rehabilitation protocols.

The most used and widely studied rehabilitation protocol is the Alfredson protocol.^64^ This protocol consists of eccentric training of the triceps surae that can be performed without supervision and minimal equipment. It consists of two sets of 15 repetitions performed twice a day, 7 days a week, for 12 weeks. The exercises include single-leg plantar flexion with the knee extended and knee flexed, emphasizing the eccentric phase of the exercise. Other protocols such as those described by Silbernagel et al., Beyer et al. and Mafi et al. proposed modifications focusing on concentric protocols, seated position, plyometrics, speed of contraction, and load during each repetition.^65^ However, there is a lack of high-quality comparative studies to prove the superiority of any single program.^65^

Current rehabilitation protocols have slight variations but are fundamentally similar. They focus on the progressive strengthening of the muscle and tendon to meet the demands imposed by the patient's body and activities.^65^ However, this approach has two main problems: (1) adherence tends to vary because pain is expected during rehabilitation,^66^ and (2) in many cases, loss of plantar flexor strength is not present, suggesting that other causal factors contributing to the onset and symptoms of AT are not being addressed.^67^ As demonstrated in the previous section of this review (Table 1), biomechanical risk factors such as reduced peak dorsiflexion, peak knee flexion and decreased forward progression of propulsion are associated with AT, possibly contributing to its onset and persistence of symptoms. If a causal link is better demonstrated in future studies, not addressing these biomechanical risk factors means not addressing important root causes of AT. Ultimately, overlooking biomechanical risk factors may compromise effective management.

Effective interventions to address biomechanical risk factors present in gait are scarce in the AT literature. A systematic review that included 27 studies examined various biomechanical parameters in runners and the effectiveness of gait modifications.^68^ Regarding the rearfoot eversion angle at initial contact, no evidence was found to support that step length manipulation can change this parameter. It was shown that peak rearfoot eversion can be increased with a crossover gait and decreased with a laterally "wide step".^68^ Also, changes in strike pattern can modify peak eversion angle. It was shown that peak eversion decreased with a forefoot strike compared to a rearfoot strike and increased with a toe strike.^68^ No significant differences were observed with a change in step length to modify peak rearfoot eversion.^68^

The systematic review by Napier et al. also assessed gait modifications that could influence knee flexion during running.^68^ There was no evidence to support that modification in stride length affected sagittal knee angle at initial contact in runners. However, the evidence for modification in foot strike was consistent across studies. Changes from rearfoot strike to forefoot strike were shown to decrease the peak rearfoot eversion. This effect was also more pronounced when toe foot strike was adopted.

In summary, there is limited evidence on the best interventions to modify the kinematic, kinetic, and neuromuscular parameters associated with AT. Therefore, we present our exercise suggestions to address the most important biomechanical variables, in case they are identified with biokinetic analysis in a clinical setting.

### Authors’ Preference of Exercises

#### Decreased Plantar flexor strength (extended knee)

The gait phase that requires more plantar flexor strength is the push-off, with soleus muscle being the major contributor to power generation.^69^ Weakness in this phase has been demonstrated previously by Kim et al.^28^ According to the principle of task specificity, we adopted this exercise (Video 1) as the standard approach to target plantar flexor strength, because it reproduces the push-off phase in the running gait cycle. The body's diagonal inclination simulates the position encountered during running, aiming to replicate the force vectors that the plantar flexors must overcome in this activity. In our practice, we train both concentric and eccentric phases with an emphasis on exercise specificity, considering the speed that is required for contraction. Progression to increased speeds on both concentric and eccentric phases is prioritized before adding additional load for the execution of the movement.

**Video 1:**
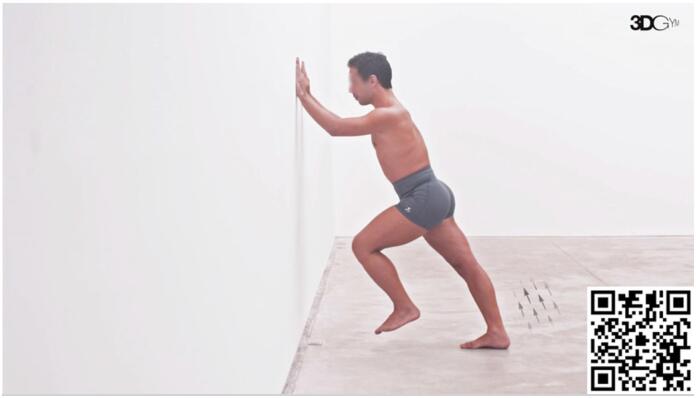
Diagonal Propulsion. Scan the QR code in the figure to view the video (https://youtu.be/iX9nPvGaCE4). Video courtesy of 3D Gym App.

Source: Video provided by 3DGym.app S.A.

In the initial phase, when pain can be a limiting factor for the proper execution of this task, our preferred approach is to increase the force sharing for plantar flexion with fibularis muscles. Fibularis muscles are secondary plantar flexors and contribute to sharing the forces required by triceps surae to produce plantar flexion. The exercise demonstrated in Video 2 depicts an elastic band inducing ankle inversion, that is counterbalanced by the action of fibularis muscles.

**Video 2:**
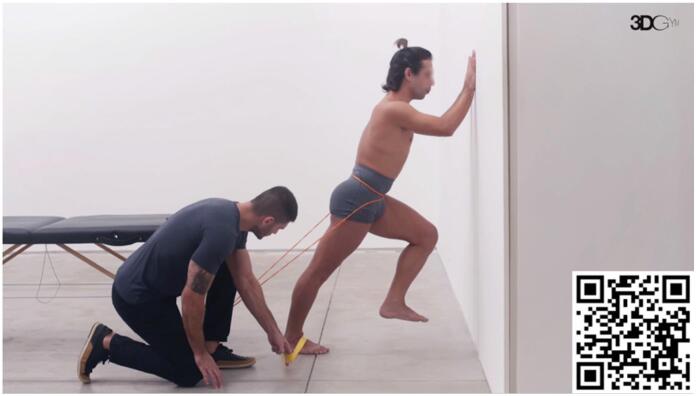
Diagonal Propulsion with Fibularis Stimulation. Scan the QR code in the figure to view the video (https://youtu.be/QtCGTSaHrcA). Video courtesy of 3D Gym App.

Source: Video provided by 3DGym.app S.A.

#### Decreased maximum ankle dorsiflexion and maximum knee flexion during gait and running

Peak ankle dorsiflexion and peak knee flexion are usually achieved during the loading response phase.^70^ In this exercise (Video 3), the individual is positioned in this phase of gait to increase the specificity of the task. Resistance is applied to the popliteus fossa and induces a concomitant hip flexion, knee flexion, and ankle dorsiflexion. The objective is to gradually prepare the individual to reach higher ankle dorsiflexion and knee flexion, training the individual to resist hip and knee flexion and ankle dorsiflexion forces in this phase of the gait. The exercise progression is made with gradual increases in applied load, speed, and number of repetitions and series as tolerated by the patient.

**Video 3:**
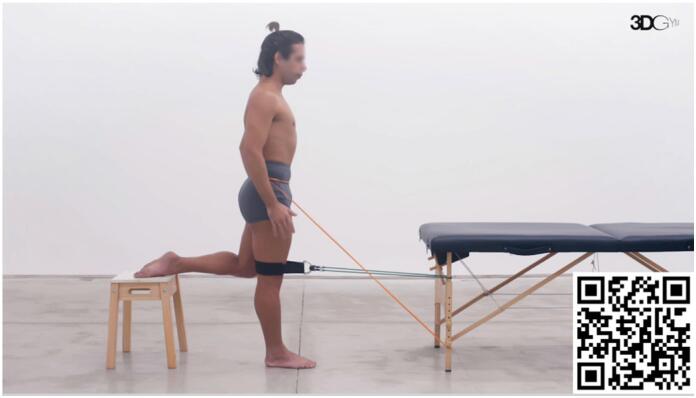
Load Response Phase Training. Scan the QR code in the figure to view the video (https://youtu.be/-_IN5Airyco). Video courtesy of 3D Gym App.

Source: Video provided by 3DGym.app S.A.

#### Increased rearfoot eversion

Peak rearfoot eversion usually occurs after heel strike, during loading response, and sometimes later in midstance.^27^ This exercise (Video 4) aims to induce an external eversion torque using an elastic band in the ankle. The expected effect is the development of a specific neuromuscular capacity to resist the external eversion stimulus in the midstance phase. The progression is made with gradual increases, as in the previous exercise.

**Video 4:**
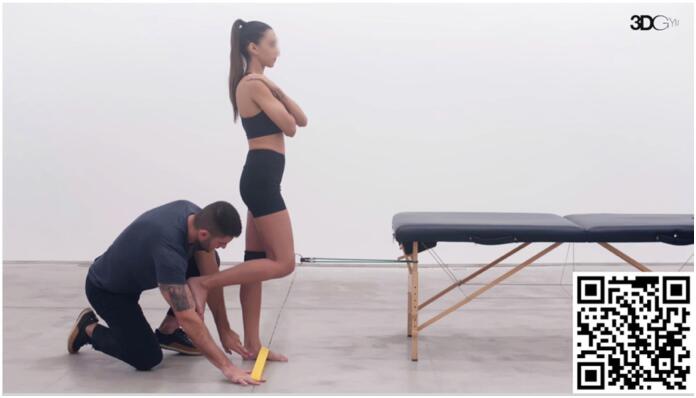
Single Leg Squat with Eversion Control with Elastic Band. Scan the QR code in the figure to view the video (https://youtu.be/1HxihUiL3x8). Video courtesy of 3D Gym App.

Source: Video provided by 3DGym.app S.A.

Excessive rear foot eversion may also be counterbalanced by increasing foot stability. The exercise in Video 5 demonstrates an elastic band under the hallux. The objective of the exercise is to induce hallux flexor contraction by preventing the band from slipping. The muscle action generates increased foot rigidity by increasing muscle stiffness and also by depressing the first metatarsus head, which increases the tension of the medial longitudinal arch.^71,72^

**Video 5:**
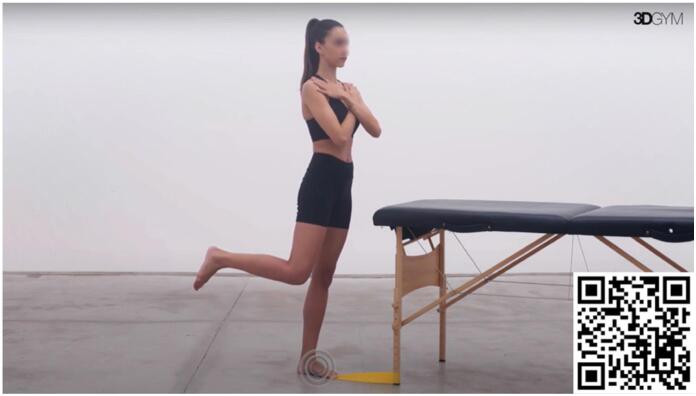
Single Leg Balance with Hallux Flexion. Scan the QR code in the figure to view the video (https://youtu.be/noUQ6dPGiXg). Video courtesy of 3D Gym App.

Source: Video provided by 3DGym.app S.A.

#### Altered gluteus complex activation

To address proximal deficiencies as demonstrated by Smith et al., who reported delayed onset and shorter duration of gluteus medius and maximus activation in runners with AT,^45^ it is possible to add an elastic band in the pelvis to induce a contralateral pelvic drop and promote the activation of the gluteus complex (Video 6). This exercise promotes the development of neuromuscular control and strengthening of the hip in synergy with the distal part of the kinetic chain, while in a position that resembles the midstance phase of gait, adding specificity to the rehabilitation.

**Video 6:**
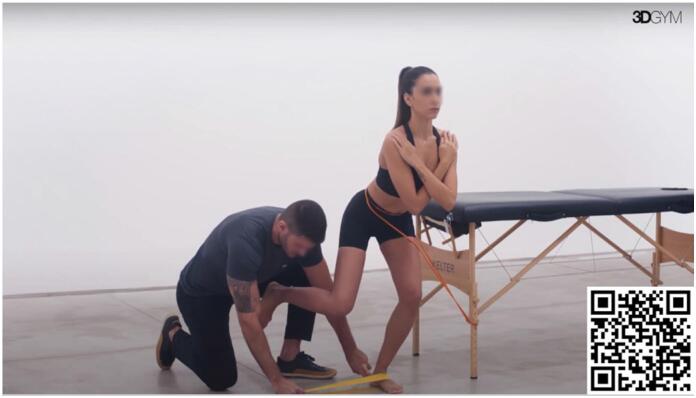
Single Leg Squat with Rearfoot Eversion Control and Contralateral Pelvic Drop Control. Scan the QR code in the figure to view the video (https://youtu.be/sM2eFq-oMec). Video courtesy of 3D Gym App.

Source: Video provided by 3DGym.app S.A.

#### Less anterior displacement of the center of pressure (COP) at push-off

Less anterior displacement of COP was interpreted by the authors to occur concomitantly to decreased knee flexion at push-off.^17^ In this exercise (Video 7), the patient is positioned in the terminal stance phase and a force is applied inferiorly and anteriorly in the proximal region of the tibia to cause knee flexion. The objective of the exercise is to develop the capacity to generate full knee extension during this specific phase of the gait cycle. Progression involves gradual increases, as in the previous exercise.

**Video 7:**
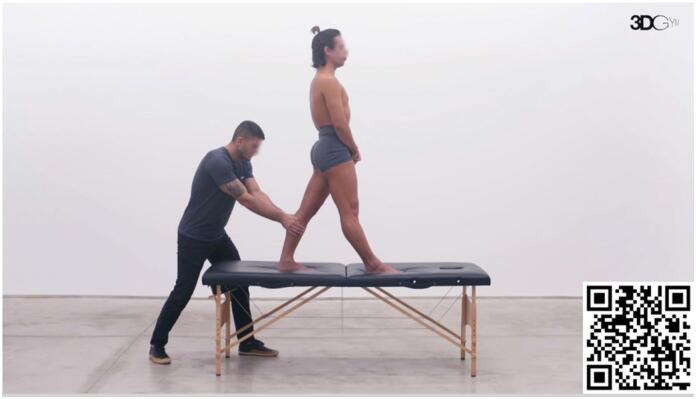
Posterior displacement of the tibia during the terminal stance phase. Scan the QR code in the figure to view the video (https://youtu.be/zzH6u-oogx8). Video courtesy of 3D Gym App.

Source: Video provided by 3DGym.app S.A.

## CONCLUSION

AT remains a prevalent and challenging condition, particularly among athletes and those involved in regular physical activities. Despite the strength and resilience of the Achilles tendon, it is highly susceptible to injury and tendinopathy due to various intrinsic and extrinsic factors. The multifactorial etiology of AT, including biomechanical risk factors like altered kinematic and kinetic parameters, highlights the complexity of effectively treating this condition. While current rehabilitation protocols, such as the widely used Alfredson protocol, focus primarily on progressive strengthening, they often overlook critical biomechanical issues contributing to the persistence and onset of AT symptoms. Comprehensive treatment strategies should incorporate targeted exercises that address specific biomechanical deficiencies, such as reduced dorsiflexion, peak knee flexion, and excessive rearfoot eversion, to enhance recovery and prevent recurrence. Integrating these biomechanical considerations into rehabilitation protocols enables clinicians to provide more effective and individualized care for patients suffering from AT, ultimately improving outcomes and quality of life.
